# Mouse nidovirus LDV infection alleviates graft versus host disease and induces type I IFN‐dependent inhibition of dendritic cells and allo‐responsive T cells

**DOI:** 10.1002/iid3.157

**Published:** 2017-04-04

**Authors:** Mélanie Gaignage, Reece G. Marillier, Catherine Uyttenhove, Nicolas Dauguet, Anubha Saxena, Bernard Ryffel, Thomas Michiels, Jean‐Paul Coutelier, Jacques Van Snick

**Affiliations:** ^1^ de Duve InstituteUniversité Catholique de LouvainBrusselsBelgium; ^2^ Ludwig Cancer ResearchBrussels BranchBrusselsBelgium; ^3^ Laboratory of Experimental and Molecular Immunology and Neurogenetics (INEM)University of OrleansOrleansFrance; ^4^ Institute of Infectious Disease and Molecular Medicine, RSAUniversity of Cape TownCape TownSouth Africa

**Keywords:** DC, GVHD, nidovirus, type I IFN

## Abstract

**Introduction:**

Viruses have developed multiple mechanisms to alter immune reactions. In 1969, it was reported that lactate dehydrogenase‐elevating virus (LDV), a single stranded positive sense mouse nidovirus, delays skin allograft rejection and inhibits spleen alterations in graft versus host disease (GVHD). As the underlying mechanisms have remained unresolved and given the need for new therapies of this disease, we reassessed the effects of the virus on GVHD and tried to uncover its mode of action.

**Methods:**

GVHD was induced by transfer of parent (B6) spleen cells to non‐infected or LDV‐infected B6D2F1 recipients. In vitro mixed‐lymhocyte culture (MLC) reactions were used to test the effects of the virus on antigen‐presenting cells (APC) and responder T cells.

**Results:**

LDV infection resulted in a threefold increase in survival rate with reduced weight loss and liver inflammation but with the establishment of permanent chimerism that correlated with decreased interleukine (IL)‐27 and interferon (IFN)γ plasma levels. Infected mice showed a transient elimination of splenic CD11b+ and CD8α+ conventional dendritic cells (cDCs) required for allogeneic CD4 and CD8 T cell responses in vitro. This drop of APC numbers was not observed with APCs derived from toll‐like receptor (TLR)7‐deficient mice. A second effect of the virus was a decreased T cell proliferation and IFNγ production during MLC without detectable changes in Foxp3+ regulatory T cell (Tregs) numbers. Both cDC and responder T cell inhibition were type I IFN dependent. Although the suppressive effects were very transient, the GVHD inhibition was long‐lasting.

**Conclusion:**

A type I IFN‐dependent suppression of DC and T cells just after donor spleen cell transplantation induces permanent chimerism and donor cell implantation in a parent to F1 spleen cell transplantation model. If this procedure can be extended to full allogeneic bone marrow transplantation, it could open new therapeutic perspectives for hematopoietic stem cell transplantation (HSCT).

## Introduction

Hematopoietic stem cell transplantation (HSCT) is still the only curative treatment for severe malignant hematologic disorders but remains hampered by graft versus host disease (GVHD). This complication is fatal in approximately 15% of transplant recipients 1, 2. GVHD also limits HSCT therapies in non‐malignant hematopoietic disorders such as Sickle cell disease 3, aplastic anemia 4, and immune deficiencies like AIDS 5.

GVHD occurs when donor T cells mount a strong immune response against the host after activation by minor and major histocompatibility antigens and cytokine storms induced by recipient conditioning 6. Antigen presentation plays a major role in the initiation of this process 7 and its inhibition, for example by blockade of costimulation, has been explored as a potential therapeutic approach of human HSCT 8. Similarly, in the mouse, inhibition of costimulation during antigen presentation was reported to limit disease 9. However, questions remain regarding the type of antigen‐presenting cells (APCs) critical for initiating the process. Host dendritic cells (DCs) are clearly potent GVHD initiators 10, 11 but non‐hematopoietic recipient APCs may also induce alloreactive donor T lymphocyte activation and acute lethal GVHD 12. At later time points, cross‐presentation by donor APCs results in persistent pathogenic T cell stimulation 13.

Infectious agents through their pathogen‐associated molecular patterns (PAMPs) and stimulation of innate pattern‐recognition receptors (PRRs) such as toll‐like receptors (TLRs) or nod‐like receptors (NLR) are significantly implicated in GVHD development. A major source of pathogens and PAMPs is initially the gastrointestinal tract but, later, microbes translocate systemically due to damage of epithelial barriers after allo‐HSCT 14. This is the case for lipopolysaccharide (LPS), a component of Gram‐negative bacteria, which is a potent TLR4 activator, contributing to severe GVHD via tumor necrosis factor‐α (TNFα) secretion. The critical involvement of LPS‐TLR4 was confirmed with an LPS antagonist that decreases GVHD severity 15, 16. In humans, colonization of intestine by *Candida* spp leads to a severe GVHD as compared to uncolonized patients 17. On the other hand, certain commensal bacteria such as *Lactobacilli* seem to play a beneficial role in mouse GVHD pathogenesis. Elimination of this species from the mouse flora before allo‐HSCT aggravates GVHD whereas its reintroduction has the opposite effect 18. Also, under certain conditions, TLR4 activation seems to have a benefic role against the disease 19. Together, these data show that environmental factors can both positively and negatively influence HSCT outcome.

In 1969, lactate dehydrogenase‐elevating virus (LDV), a single stranded positive‐sense RNA enveloped mouse nidovirus 20, was reported to prolong skin allograft survival and to inhibit spleen size changes in a parent to F1 non‐irradiated GVHD model 21. However, no data were provided on the effect of the virus on final GVHD outcome and mechanistic analysis was of course limited by the available technology. To the best of our knowledge, no attempt to better characterize the effects of LDV in GVHD has been reported since.

Given the interest in unraveling novel GVHD prevention mechanisms, we readdressed the effect of LDV infection in the B6 > B6D2F1 parent to F1 acute GVHD model. This model was selected to fit the conditions used in the above‐mentioned publication and also because it focuses on the effects of a viral infection on the allo‐immune reaction in the absence of the cytokine storm resulting from host irradiation. We observed that LDV confers significant long lasting protection in this GVHD model, leading to the establishment of chimerism associated with diminished interleukine (IL)‐27 and interferon (IFN)γ production as well as an impaired conventional DC function that depended on TLR7 and type I IFN signaling. Transient suppression of allogeneic T cell responsiveness was also observed. These results show that a short timely inhibition of DC and donor T cell allo‐responsiveness resulting in impaired IFNγ and IL‐27 production may provide long lasting protection against GVHD.

## Results

### LDV infection prevents acute B6 to B6D2F1 GVHD mortality and morbidity

**Figure 1:**
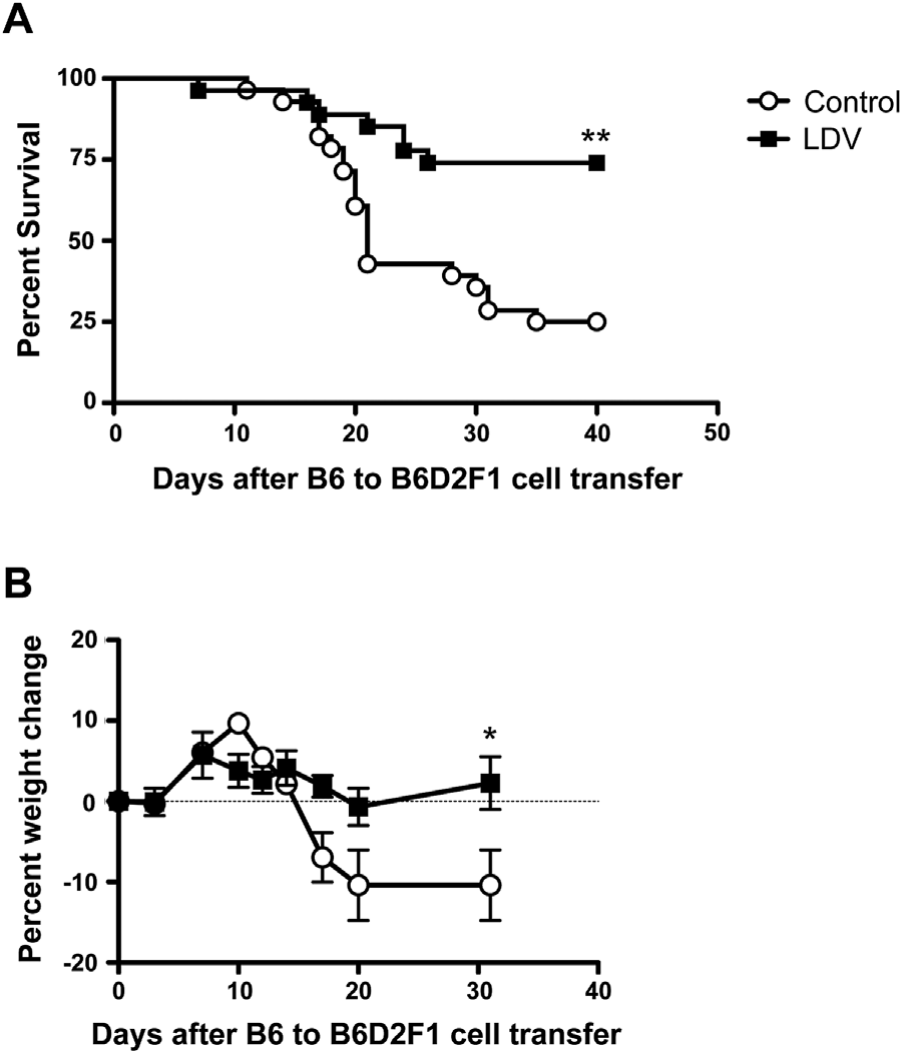
LDV infection protects mice against aGVHD. B6D2F1 recipient mice were infected or not with LDV 24 h before transfer of 60 × 10^6^ B6 splenocytes. (A) Mice were monitored for mortality. Data are from five pooled experiments with a total of 28 control and 27 infected mice (**P < 0.01 Log‐rank (Mantel‐Cox) Test). (B) Mice were monitored for weight loss. Data are means ± SEM (*n* = 5 mice per group) of one experiment and representative of three (*P < 0.05 by Anova–Bonferoni post‐test).

We tested the effect of LDV infection on acute GVHD (aGVHD) induced in B6D2F1 recipients of B6 spleen cells. Infection of recipient mice with LDV 24 h before B6 cell transfer conferred significant protection against disease. In pooled data of five experiments involving a total of 28 control and 27 infected mice (Fig. 1A), mortality was significantly decreased after infection, dropping from 75% in control to 25% in LDV‐infected animals. Moreover, weight loss, a marker of morbidity in mouse aGVHD, was completely suppressed in the infected survivors (Fig. 1B).

### LDV infection inhibits IFNγ and IL‐27 production and prevents liver and spleen damages

**Figure 2:**
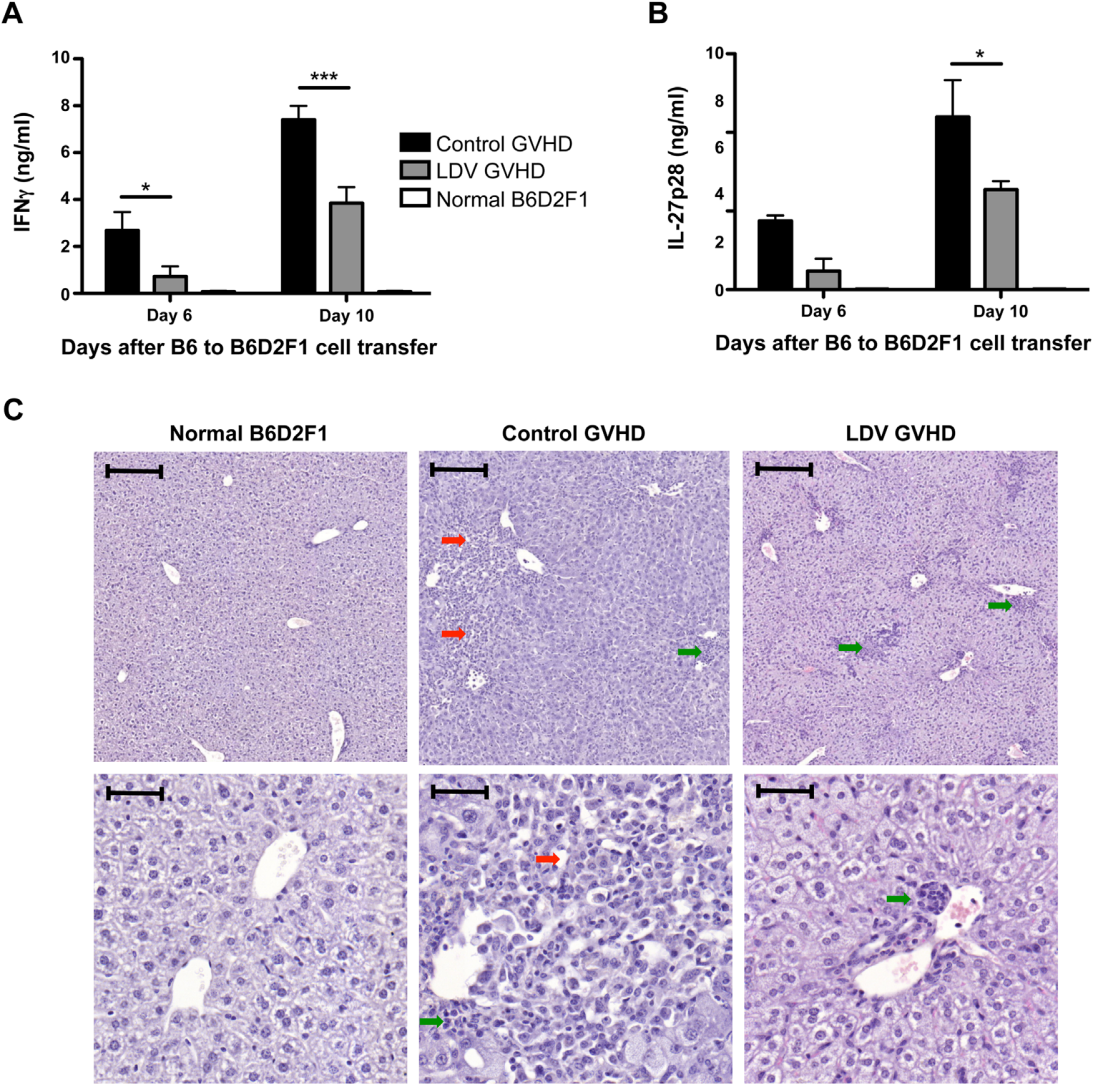
LDV infection lowers plasma inflammatory cytokine levels and abrogates liver lesions. B6D2F1 mice were infected or not with LDV 24 h before transfer of 60 × 10^6^ B6 spleen cells. (A) IFNγ and (B) IL‐27p28 were measured by ELISA in plasma prepared from mice bled on days 6 and 10. Data are means ± SEM (*n* = 3 mice per group for day 6 and *n* = 5 mice per group for day 10) and representative of two independent experiments (*P < 0.05, **P < 0.01, ***P < 0.001 by 2 way ANOVA and Bonferroni post‐test). (C) Liver sections were prepared from normal B6D2F1 or control or LDV mice at day 16 of GVHD. Mononuclear inflammatory cells present in portal tracts (green arrow) and large‐dilated sinusoidal spaces (red arrow) are indicated. Scale bars in upper and lower panels represent 200 and 50 µm, respectively. Slides are representative of two independent experiments with five mice per group.

According to previous work 22, in the B6 to B6D2F1 model of GVHD, IFNγ and IL‐27 are good markers of acute GVHD that peak shortly after allogeneic cell transfer. We therefore measured these cytokines in the serum six and 10 days after cell transfer. LDV infection significantly decreased IFNγ and IL‐27p28 levels in the serum compared to control GVHD mice (Fig. 2A and B).

Liver tissue damage is an important marker of acute GVHD. Histological analysis was therefore performed on liver sections between 14 and 18 days after B6 spleen cell transfer to B6D2F1 mice (depending on disease severity). Large lesions were found in the livers of control mice that received B6 spleen cells but not in LDV‐infected mice. However, LDV infection did not prevent mononuclear cell infiltration as illustrated in the representative micrograph shown in Figure 2C.

### LDV infection allows establishment of donor‐host splenic chimerism

**Figure 3:**
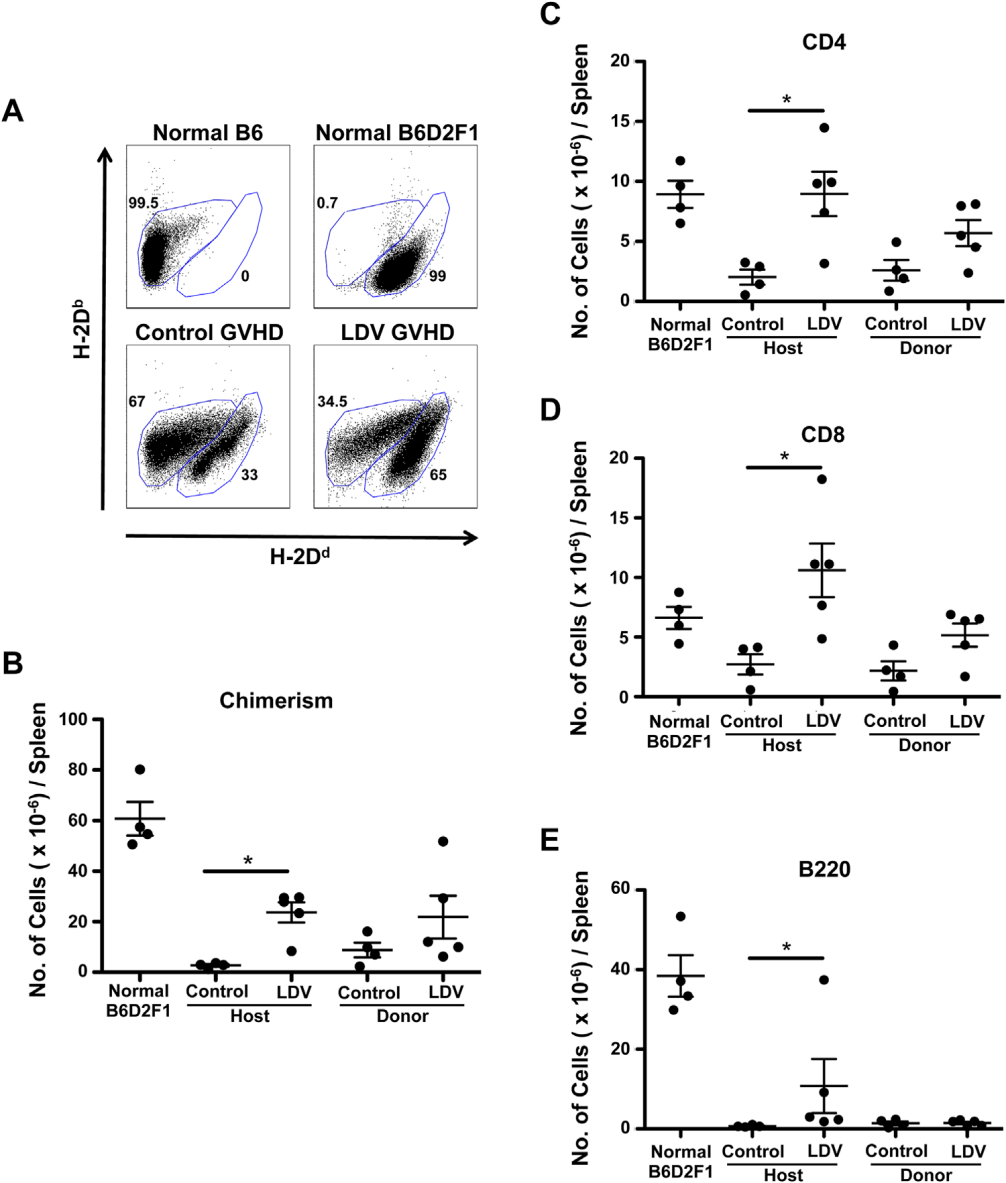
LDV infection prevents host cell destruction. B6D2F1 recipient mice were infected or not with LDV 24 h before transfer of 60 × 10^6^ B6 spleen cells (A and B). Fourteen days after B6 spleen cell transfer, anti‐H‐2D^d^ FITC and H‐2D^b^ PE were used to analyze the presence of host and donor cells by flow cytometry. (A) Representative dot plots illustrate percentages (gated on total living cells) and (B) absolute numbers of host (H‐2D^d‐b^) and donor (H‐2D^b^) cells. Data are means ± SEM (*n* = 4–5 mice per group) and are representative of three independent experiments (*P < 0.05 Kruskal–Wallis test with Dunn's Multiple Comparison Test). (C–E) Fourteen days after B6 spleen cell transfer, spleen cell subsets were enumerated by FACS, using anti‐H2Dd–FITC, (C) anti‐CD4, (D) anti‐CD8, and (E) anti‐B220. Data in all panels are means ± SEM (*n* = 4–5 mice per group) and are representative of three independent experiments (*P < 0.05 (Mann–Whitney unpaired *t*‐test).

To determine whether B6 splenocytes were able to engraft in LDV‐infected mice, we labeled B6D2F1 spleen cells 14 days after B6 spleen cell transfer with anti‐MHC class‐I haplotype antibodies (anti‐H‐2D^d−^D^b+^ for B6 and anti‐H‐2D^d+^D^b+^ for B6D2F1) (Fig. 3A). Flow cytometry analysis indicated that donor cells were present in the spleen of infected mice and that their numbers increased as compared to non‐infected recipients but the difference did not reach statistical significance (8.8 ± 3.6 × 10^6^ in controls and 21.8 ± 8.5 × 10^6^ in LDV mice [*P *> 0.05]) (Fig. 3B). A similar trend was seen for CD4 and CD8 T cells but B cells were barely detectable (Fig. 3C–E). On the other hand, host spleen cell survival was dramatically increased in infected mice (LDV: 23.7 ± 4.1 × 10^6^ vs. control GVHD 2.7 ± 0.7 × 10^6^. *P* < 0.01 as compared to 60.7 ± 6.8 10^6^ in normal B6D2F1 mice). In fact, the destruction of host CD4 and CD8 T cells was completely inhibited by LDV while that of host B cells was only partially but significantly diminished (Fig. 3C–E). Thus, LDV did not at all impair donor T cell implantation but inhibited host B and T cell depletion.

### LDV infection inhibits both CD4 and CD8 T cell stimulation

**Figure 4:**
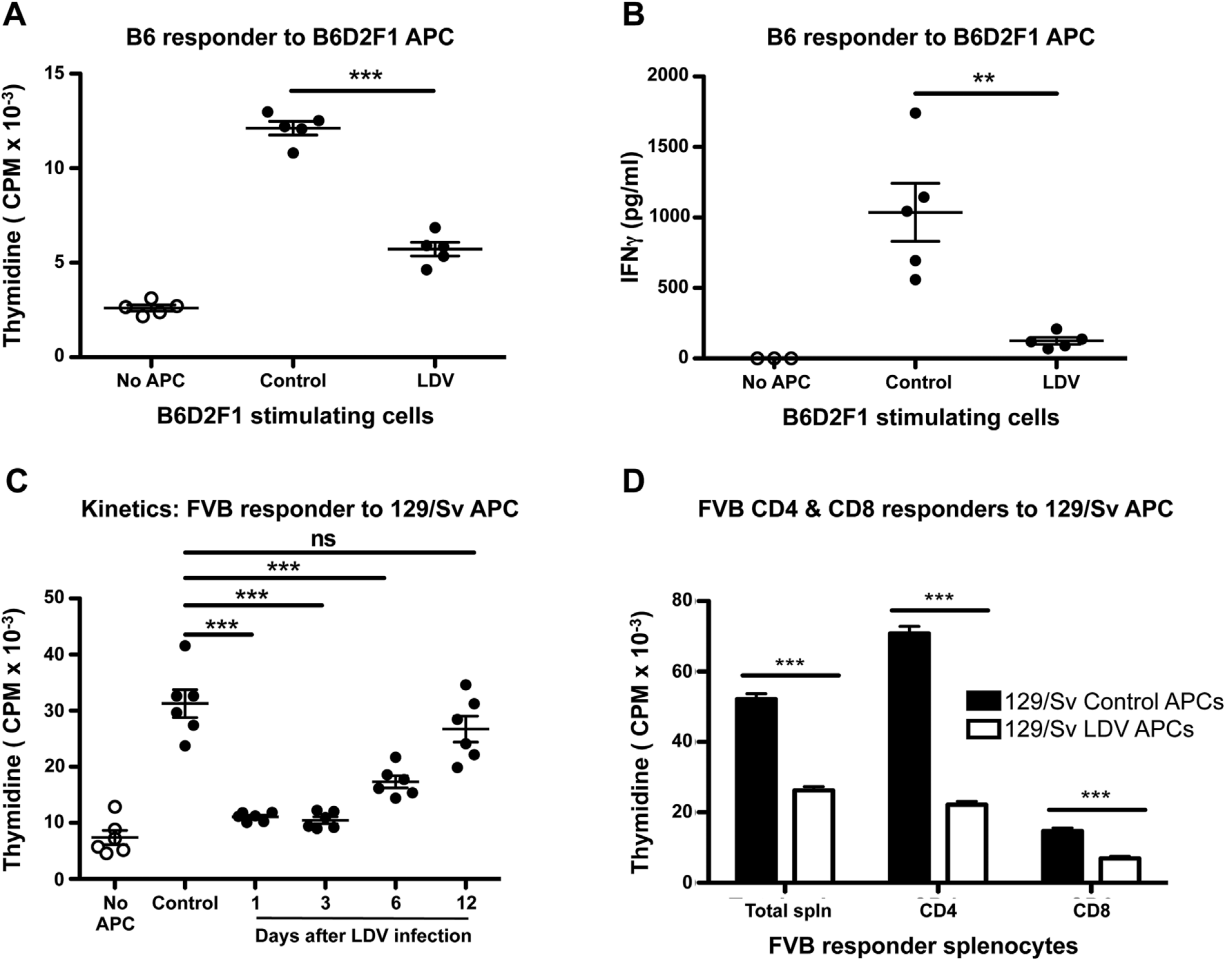
LDV‐infection transiently inhibits in vitro class I and II allo‐antigen responses. Splenic APCs from LDV infected (72 h) and non‐infected B6D2F1 mice were irradiated and incubated with normal B6 spleen cells. After 48 h, (A) proliferation and (B) IFNγ production were determined by 3H‐thymidine incorporation and ELISA, respectively. Data are means ± SEM (*n* = 5 mice per group) and representative of three independent experiments. (**P < 0.01, ***P < 0.001 (Student and Mann–Whitney *t*‐test) (C) Infection kinetics: 129/Sv‐APCs were recovered at various time points after LDV infection and incubated with FVB responder spleen cells for 48 h. The proliferation was measured by 3H‐thymidine incorporation. Data are means ± SEM of two pooled experiments and representative of four independent experiments (***P < 0.001, One‐way analysis of variance with Bonferroni's Multiple Comparison Test). (D) APCs from LDV infected and non‐infected 129/Sv mice were recovered after 24 h, incubated with total, CD4+ or CD8+ spleen cells from FVB mice. Cells were pulsed with 3H‐thymidine for 18 h to determine proliferation. Data are means ± SEM (*n* = 5 mice per group) and representative of three independent experiments (***P < 0.001 Two‐way ANOVA with Bonferroni post‐tests).

LDV has been reported to impair MHC class II‐mediated protein antigen presentation to in vivo primed T lymphocytes 23. To determine whether this mechanism also operates in allogeneic reactions, we tested the influence of the virus on allogeneic mixed‐lymphocyte‐culture (MLC). B6D2F1 (H‐2D^b/d^) mice were infected or not with LDV 72 h before co‐culture of their adherent spleen cells with normal B6 responder splenocytes (H‐2D^b^). Both proliferation and IFNγ production were strongly inhibited if stimulator cells were collected 72 h after LDV infection (Fig. 4A and B). Similar observations were made in other fully allogeneic reactions such as FVB (H‐2^q^) > 129/Sv (H‐2^b^) (Fig. 4C), CBA (H‐2^k^) > 129/Sv, or B6 > BALB/c (H‐2^d^) (data not shown). Of note, any action on the responder cells of traces of the virus remaining in the APC preparation is ruled out by the short survival time of the virus in vitro 24.

The kinetics of LDV‐mediated suppression was tested in the FVB > 129/Sv combination. Abrogation of the response was maximal if stimulator cells were prepared from spleens collected one or three days after infection. Both proliferation (and IFNγ production, data not shown) started to recover after six days and returned to normal after 12 days (Fig. 4C).

The suppression of allogeneic reactions by LDV described in the allogeneic MLC experiments suggests that both class I and II antigenic reactions were suppressed. To directly address this question, adherent spleen cells from 129/Sv mice (H‐2^b^) were incubated with purified CD4^+^ or CD8^+^ T cells (anti‐MHC II Ab was added to inhibit background MHC II reactions in CD8 experiments) from FVB (H‐2^q^) spleens. Total splenocytes, purified CD4^+^ and CD8^+^ responder cells incubated with APCs from LDV‐infected mice all had significantly reduced proliferation compared to responder cells incubated with APCs from non‐infected mice (Fig. 4D). Together, the data indicate that LDV infection transiently affects splenocyte APCs that become unable to stimulate allogeneic CD4^+^ and CD8^+^ T cells.

### LDV inhibits cDC stimulatory activity of allogeneic spleen cells in vitro

**Figure 5:**
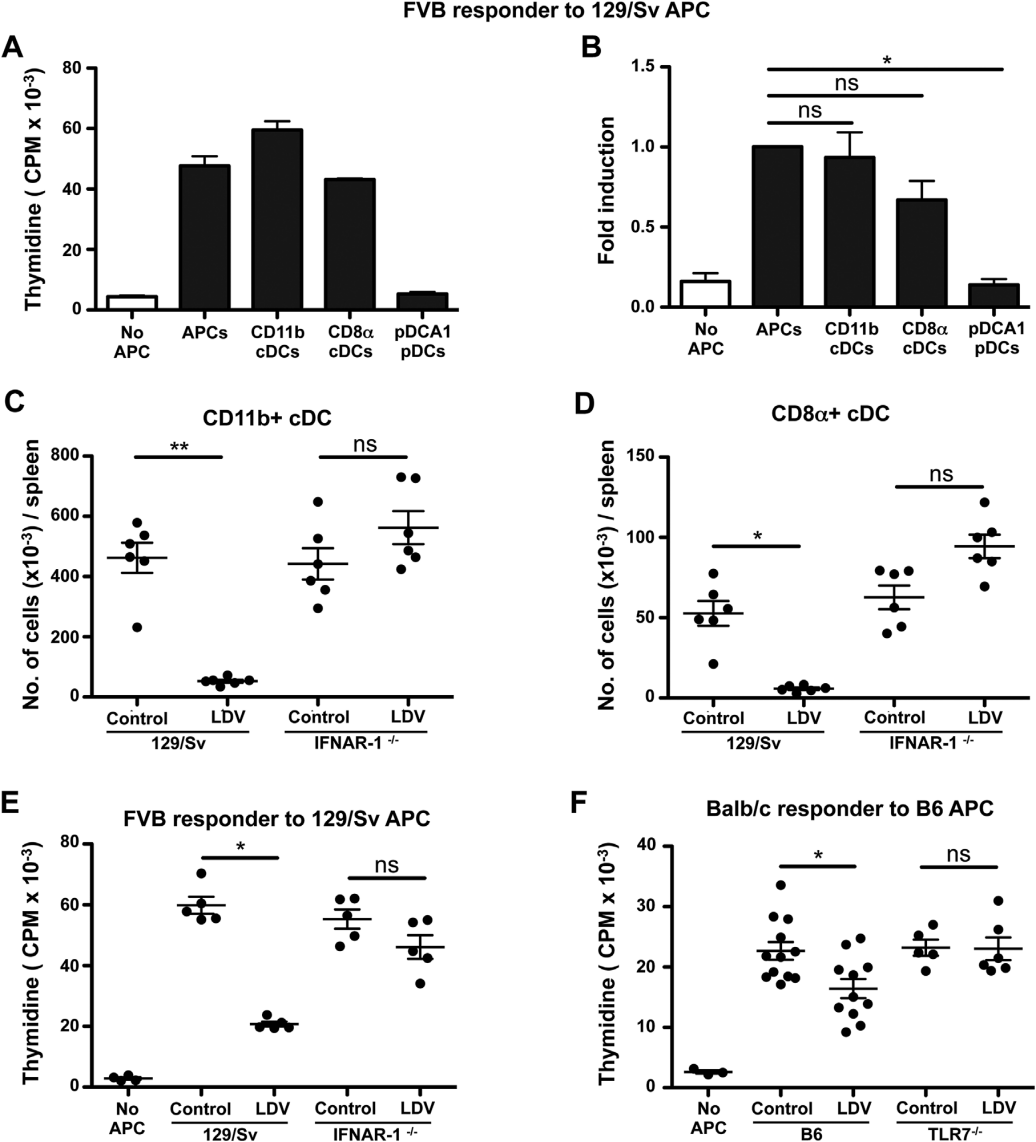
LDV‐infection affects conventional dendritic cell stimulation of allogeneic MLC in a type I IFN‐ and TLR7‐dependent process. (A and B) FVB (H2^q^) spleen cells were incubated without APC or with CD11b+ cDCs, CD8α+ cDCs or pDCs purified by MACS beads and FACS sorting from normal 129/Sv mice. After 48 h, proliferation was measured by 3H‐thymidine incorporation. (A) Representative experiment (mean ± SD of experimental triplicates). (B) Fold induction compared to splenocytes. Data are pooled of three independent experiments (*P < 0.05, Kruskal–Wallis test with Dunn's Multiple Comparison Test). (C and D) Spleen cells from non‐infected and three day LDV‐infected 129/Sv or 129‐IFNAR‐1^−/−^ mice were labeled with anti‐CD11c, anti‐B220, anti‐MHCII, anti‐CD11b, and anti‐CD8 Abs and analyzed by FACS. The total number of spleen CD11b+ cDCs and CD8α+ cDCs are represented by dot plots. Data are means ± SEM (*n* = 6 mice per group) and representative of four independent experiments (*P < 0.05, **P < 0.01, Kruskal–Wallis test with Dunn's Multiple Comparison Test). (E) Type I IFN signaling is required for LDV suppressive activity. Splenic APC from one day LDV‐infected and non‐infected 129/Sv WT or 129‐IFNAR‐1^−/−^ mice were incubated with normal FVB spleen cells. The proliferation was measured by 3H‐thymidine incorporation. Data are means ± SEM (*n* = 5 mice per group) and representative of three independent experiments (*P < 0.05, Kruskal–Wallis test with Dunn's Multiple Comparison Test). (F) TLR7 activation is required for LDV suppressive activity. APC from one day LDV‐infected and non‐infected B6 WT or B6 TLR7^−/−^ mice were isolated from the spleens and incubated with normal FVB spleen cells. The proliferation was measured by 3H‐thymidine incorporation. Data are means ± SEM of two pooled experiments (*n* = 5–12 mice per group) representative of three experiments (*P < 0.05, Kruskal–Wallis test with Dunn's Multiple Comparison Test).

To analyze the mechanisms underlying the suppressive effects of LDV on allogeneic Ag stimulation, we first identified the allogeneic Ag presenting spleen cells responsible for T cell activation in vitro. Initial analysis showed that CD11c^+^ cells but not CD11b^+^CD11c^−^ cells (mainly macrophages) stimulated allogeneic T lymphocytes (Supplementary Fig. S1A). Further characterization of the allogeneic stimulating APC population performed by FACS sorting (Supplementary Fig. S1B) indicated that CD11c^+^‐MHC II^+^‐B220^−^‐CD11b^+^ and CD11c^+^‐MHC II^+^‐B220^−^‐CD8α^+^ conventional DCs (cDCs) but not CD11c^+^‐MHC II^low^‐B220^+^ pDCA1^+^ pDCs were effective stimulators of allogeneic responder spleen cells (Fig. 5A and C). Quantification of splenic CD11b^+^ and CD8α^+^ cDCs from control or LDV‐infected mice showed that both were severely diminished after infection (CD11b^+^: 4.3 ± 0.3 vs. 0.53 ± 0.03 × 10^5^ and CD8α^+^: 5.3 ± 0.7 vs. 0.58 ± 0.09 × 10^4^) (Fig. 5C and D).

To evaluate whether this cell loss was due to lytic infection or to soluble factors like type I IFNs, which have been reported to be strongly induced early after infection 25, we repeated the infection in 129/Sv H2^b^ wild type or 129/Sv‐IFNAR‐1^−/−^ mice and observed that, in IFNAR‐1^−/−^ mice, LDV did neither decrease the number of CD11b^+^ and CD8α^+^ cDCs (Fig. 5C and D) nor their ability to function as APC in MLC reactions using FVB (H2^q^) responder spleen cells (Fig. 5E). Of note, 129/Sv‐IFNAR‐1^−/−^ mice were not resistant to LDV infection as their lactate dehydrogenase serum titer was similar to that of control 129/Sv‐infected mice (data not shown).

LDV was reported to induce TLR7 activation 26. We verified that TLR7 was required for the suppression of allo‐antigen stimulation by incubating LDV‐infected or control spleen cells from wild type or TLR7^−/−^ B6 mice with BALB/c (H2^d^) responder spleen cells. Although the LDV suppressive effect on B6 APCs was not as strong as in 129/Sv APCs, we observed that the APC function of TLR7^−/−^, contrary to that of wild‐type mice, was not affected by LDV (Fig. 5F). In addition, cDC numbers were not decreased in TLR7^−/−^ B6 spleens compared to LDV‐infected WT B6 (Supplementary Fig. S2). These results suggest that only conventional DCs are able to activate allogeneic T cell response in vitro and, following LDV‐infection, splenic cDCs are eliminated. This effect is type I IFN and TLR7 dependent.

### LDV infection decreases the reactivity of allogeneic responder T cells

**Figure 6:**
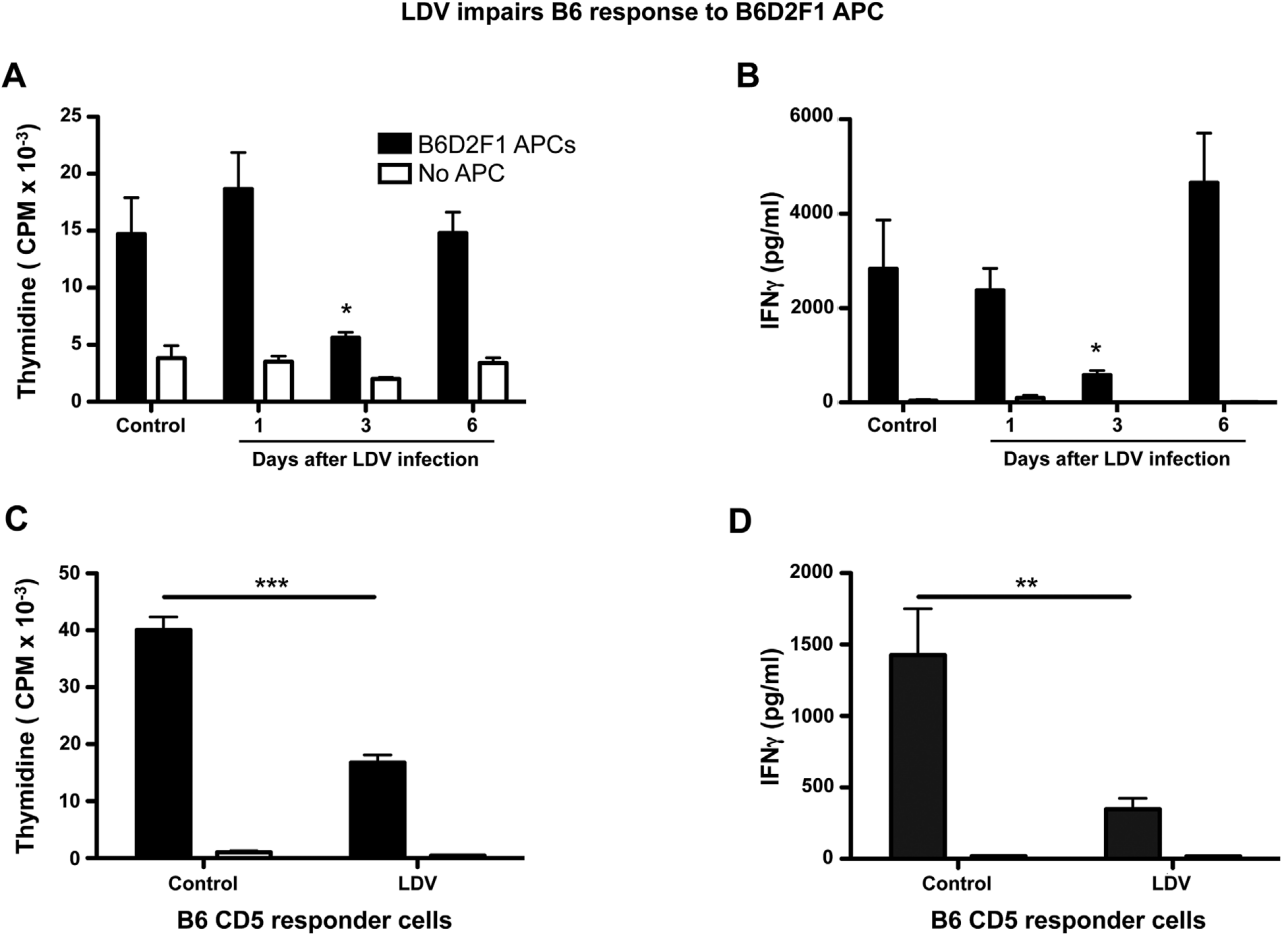
LDV causes transient hyporesponsiveness in CD5+ spleen cells three days after infection. Infection kinetics: B6 spleen cells were recovered at various time points after LDV infection and incubated with B6D2F1 irradiated adherent stimulating cells for 48 h. (A) Cells were pulsed with 3H‐thymidine for 18 h to determine proliferation. (B) After 72 h, IFNγ production was determined by ELISA. Data are means ± SEM (*n* = 5 mice per group) and representative of three independent experiments (*P < 0.05, 2 way Anova and Bonferroni post‐tests). (C and D) The CD5+ B6 cell reactivity is affected after LDV infection. B6 responder spleen cells were recovered three days after LDV infection and CD5+ cells were purified by MACS before incubation with B6D2F1 adherent stimulating cells for 48 h. After 72 h, (C) proliferation and (D) IFNγ production were determined. Data are means ± SEM of two pooled experiments (*n* = 8 mice per group) representative of three experiments (**P < 0.01, ***P < 0.001 [student and Mann–Whitney *t*‐test]).

It is well established that if the allogeneic T cells are removed from donor graft, GVHD will not be induced 27, 28. Therefore we investigated the influence of LDV on the responder component of GVHD. B6 mice were infected at several time points before testing the response of their spleen cells to non‐infected B6D2F1 APCs in vitro. These experiments showed a significant decrease in proliferation and IFNγ production that was, however, only transient (at day 3 not at day 1 or 6) (Fig. 6A and B).

To formally prove that LDV had actually modified the responder T cells, we purified splenic CD5^+^ T cells (to include both CD4 and CD8 T cells) from control or LDV‐infected B6 mice by positive selection and tested their allo‐reactivity in MLC. If the T cells were collected three days after LDV infection, both proliferation and IFNγ production were strongly inhibited (Fig. 6C and D).

**Figure 7:**
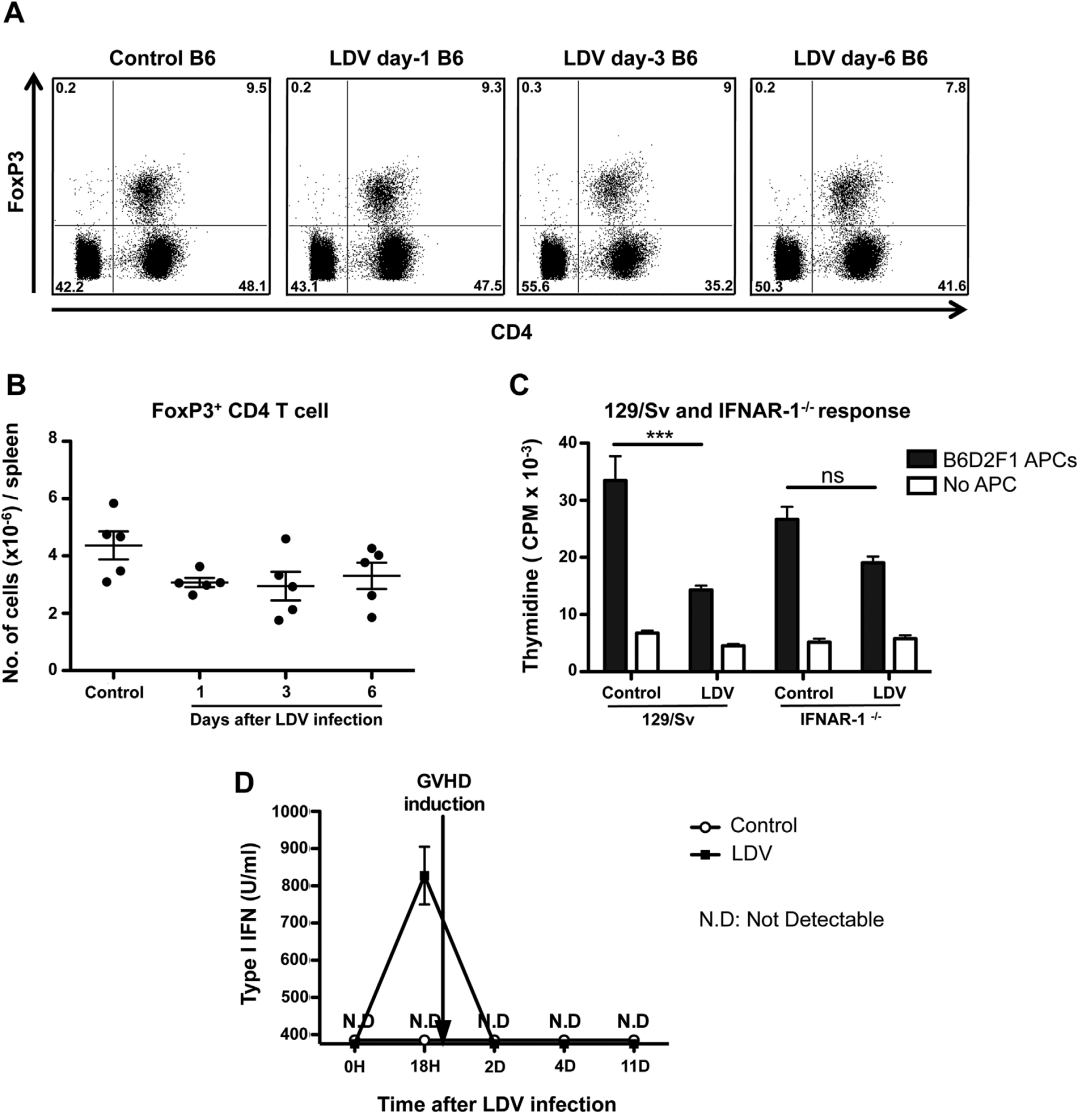
The LDV suppressive effect on T cells is not related to a change in Treg number but is IFNAR dependant. (A and B) B6 spleen cells from LDV‐infected and non‐infected mice were recovered and stained with anti‐TCRß, anti‐CD4, anti‐FoxP3, and LIVE/DEAD^®^ to determine the total number of Treg cells by FACS. Data are means ± SEM (*n* = 5 mice per group) and representative of two independent experiments. (A) Representative density plots with the percentage of each gated population. (B) The total number of FoxP3+ CD4+ TCRβ+ from spleen are represented by scatter plots. (C) The suppression of responder cells is type I IFN dependent. Responder spleen cells from three day LDV‐infected and non‐infected 129/Sv WT (*n* = 6) or 129/Sv‐IFNAR‐1^−/−^ (*n* = 6) mice were recovered and co‐cultured with normal B6D2F1 APCs. Cells were pulsed with 3H‐thymidine for 18 h to determine proliferation. Data are means ± SEM and representative of three independent experiments (***P < 0.001, Kruskal–Wallis test with Dunn's Multiple Comparison Test). (D) B6D2F1 mice were infected or not with LDV at day 0. After 24 h, 60 × 10^6^ B6 spleen cells were injected i.p. to induce GVHD. Type I IFN activity was measured in plasma from mice bled at several time points and was quantified, as described previously, by a standard cytopathic effect reduction assay performed in 96‐well plates, on BALB/3T3 cells infected with Mengo virus 46. The detection limit in plasma was ±375 units/ml (corresponding to 10 pg/ml of reference recombinant IFNα). Data are means ± SEM (*n* = 4 mice per group).

To evaluate the involvement of regulatory T cells (Tregs) in the LDV suppressive process, we analyzed the number of Tregs in spleens, of normal and LDV‐infected B6 mice at various time points using FoxP3 staining and FACS analysis. No change in percentage or absolute number was observed in Foxp3^+^ CD4^+^ T cells from one to six days after infection (Fig. 7A and B).

To test the implication of type I IFN signaling in the T cell inhibition, we infected 129/Sv or 129/Sv‐IFNAR‐1^−/−^ mice and compared the proliferation of their spleen cells to that of controls in MLC with irradiated allogeneic adherent B6D2F1 spleen cells. This experiment confirmed the inhibitory effect of LDV on the allogeneic responder cells in a different strain combination and showed that, in 129/Sv‐IFNAR‐1^−/−^ responder cells, a slight but non‐significant inhibition was observed suggesting that type I IFN plays a major role in T cell inhibition by LDV but that part of the inhibition could be IFNAR‐1 independent (Fig. 7C).

Finally, given the critical role of IFNAR‐1 in the effect of LDV on cDC suppression and T cell allo‐unresponsiveness, it was of interest to evaluate the presence of type I IFNs in infected and control GVHD mice at different time points. Figure 7D shows that type I IFN production occurred in a sharp peak 18 h after infection in accordance with previously reported data 25. This fits well with the very transient nature of DC and T cell alterations by LDV. Of note, type I IFN seems to be induced only by LDV infection and not by GVHD since one day after donor cell transplantation type I IFN was not detectable anymore. This implies that the donor cells were only shortly exposed to an IFN‐rich environment but this was sufficient to impair their allogeneic response.

## Discussion

It has been suggested that viral infections may in some circumstances predispose recipients of HSCT to severe GVHD 29. However, increasing evidence indicates that infectious agents may also prevent this adverse reaction through their interaction with innate receptors 30, 31. We used LDV as a tool to modulate the immune microenvironment during acute GVHD in a parent to non‐conditioned F1 recipient model. We selected this model as an immunologic tool to avoid the massive inflammation induced by recipient irradiation so that the effect of the virus, if any, would be easier to analyze. This relatively mild GVHD, which nevertheless killed 75% of the recipients, was partially impaired by the infection, thus further extending the suppressive effects of this virus previously reported in various auto‐immune diseases 32, 33, 34.

The protection conferred by LDV correlated with a short (±one week) inhibition of both allo‐Ag presentation by DC and donor T cell responsiveness. Allogeneic T cell stimulation by spleen cells of LDV‐infected wild type, but not TLR‐7‐deficient, mice was severely compromised after infection which coincided with selective elimination of CD11b^+^ and CD8α^+^ cDCs from the spleen. LDV infection did not prevent donor cell implantation but inhibited weight loss, host lymphocyte destruction, liver damage as well as IFNγ and IL‐27 production, two cytokines contributing to the pathology of parent to F1 GVHD 22, 35.

Our results confirm and expand data reported by Isakov who observed that LDV impaired presentation of a protein Ag by peritoneal macrophages without affecting their phagocytic activity 23. In retrospect, these peritoneal cells were probably DC because we confirmed these observation with ovalbumin presentation and found that DC, not macrophages, were the main antigen presenting cells in this system (Gaignage, unpublished observations). We here extended these results to allogeneic responses and identified CD11b^+^ and CD8α^+^ cDCs as the main initiators of these responses contrary to macrophages that were very poor APCs in MLC reactions. Of note, LDV also induced partial disappearance of CD11c^+^‐MHCII^low^‐B220^+^‐pDCA1^+^ pDC but these cells were unable to induce allogeneic T cell responses in vitro, in agreement with published data 36. Together, these results suggest that the impaired antigenic stimulation during LDV infection results from a selective suppression of cDC function providing further evidence for the critical role of these cells in GVHD, as originally reported by Shlomchik 37. Of note, although the number of cDC completely normalized two weeks after infection, donor T cells persisted without inducing a delayed GVHD reaction. Moreover, LDV infection performed one week after B6 spleen cell transplantation no longer impaired GVHD 38 further supporting the action of the virus on the initiating steps of the response.

The suppressive effect of LDV on APCs was TLR7 dependent in agreement with the reported activation of TLR7 by the virus 26 and the impairment of DC differentiation and maturation by TLR7 agonist Resiquimod 39. Given that TLR7 agonist increases IFN‐β production 40, and that APCs from IFNAR‐1^−/−^ mice were not affected by LDV, the TLR7‐type I IFN pathway seems to play a central role in the suppressive effect of LDV on allo‐Ag presentation. Of note, type I IFN activity in plasma peaked at 18 h after infection which fits well with the timing of DC cell depletion.

The above results suggested that inhibition of APCs could explain the protective effect of LDV in GVHD. However, severe depletion of host cDCs does not prevent GVHD as reported in CD11c‐DTR transgenic recipients where host cDCs were depleted by diphtheria toxin 41. Based on these observations, the protective effect of LDV was probably not just due to suppression of cDCs. In fact, CD5^+^ T cells from LDV‐infected donor mice also showed poor proliferation and IFNγ production in MLC. This inhibition was also partly dependent on type I IFN, in line with the reported exacerbation of GVHD in IFNAR‐1^−/−^ B6 recipients of BALB/c CD4 T cells and by the inhibition of allogeneic donor T cell responses by IFNα 42. However, other mechanisms may still be implicated as T cell inhibition by LDV was not completely abrogated in IFNAR‐1^−/−^ mice.

Finally, we confirmed the early and brief presence of type I IFN activity in LDV‐infected mice described earlier 25 and showed that it was not prolonged by GVHD induction. In view of the requirement of IFNAR‐1 for LDV suppressive effects in allogeneic reactions, it follows that very short exposure to type I IFNs could have profound effects on GVHD outcome.

An explanation of the protective effects of LDV infection on GVHD would thus be that massive viral replication, which peaks within the first 20 h after infection, induces strong viral RNA‐mediated activation of TLR7 which in turn elicits a strong but very transient production of type 1 IFN which affects antigen presentation by cDC and partly T cell alloresponsiveness. These changes last only a few days, suggesting that critical events occur shortly after HSCT and that their alteration can have long lasting consequences on GVHD outcome. This observation could open new perspectives for the design of GVHD prevention protocols.

## Materials and Methods

### Mice

Most mice were bred under SPF conditions at the animal facility of the Ludwig Institute Brussels Branch under the direction of Guy Warnier and Gilles Gaudray (DVM). Experimental protocols and animal handling were approved by the ethical committee of the Medical Faculty of the Université de Louvain (accreditation no: 2011/UCL/MD/014). IFN‐α/βR^−/−^ 129/Sv mice (IFNAR‐1^−/−^) were a gift of Dr. M. Aguet 43. TLR7^−/−^ 44 were provided by and maintained in the animal facility of the CNRS Orléans. LDV, Riley strain, originally obtained from the ATCC, was maintained in our laboratory by passage in NMRI mice. Infection was performed by intra‐peritoneal injection of a 2 × 10^7^ infectious dose 50 (ID50) of LDV.

### Induction of GVHD

GVHD was induced by i.p. injection of 50–70 million C57Bl/6 (B6) spleen cells in B6xDBA/2 F1 (B6D2F1) recipients one day after LDV infection. All experiments were performed on adult mice with a weight of ±20 g. Mice were monitored for survival and weight loss every other day. Mice were bled on the indicated days to monitor chimerism and serum cytokines and at the experimental endpoint, euthanized for spleen and liver analyses.

### In vitro culture

MLC was carried out by incubating 1.25 × 10^6^ responder spleen cells/ml with an equal number of irradiated (30 Gy from a 137 Cs source) adherent spleen cells. In some cases, responders were CD4^+^, CD8^+^, and CD5^+^ cells purified from spleens and seeded at a density of 0.75 × 10^6^ cells/ml. Adherent cells were obtained by coating 1 × 10^6^ splenocytes in a 96‐well flat bottom microtiterplate for 1.5 h and removing non‐adherent cells by washing the microplate twice with PBS (37°C). DC subpopulations were cultured at 10^4^ cells per well. Proliferation was measured after two days by incubation with 3H‐thymidine at 1 µCi (0.037 MBq)/well for a further 18 h. 3H‐thymidine incorporation was measured using a scintillation counter (Packard Microplate Scintillation Counter). MHC II activity was blocked in vitro using anti‐MHC II LEAF anti‐mouse I‐A/I‐E clone M5/114.15.2 (Biolegend, San Diego, CA) at 5 µg/ml.

### Flow cytometry and cell sorting

To determine chimerism, splenocytes were stained with anti‐H‐2D^d^‐FITC (clone: 34–2–12) and anti‐H‐2D^b^‐PE (clone: KH95) (all from Biolegend). Treg cells were determined in the spleens according to manufacturer's instructions using a kit from eBioscience (San Diego, CA). Spleen cells were also characterized using anti‐CD11b (clone: M1/70), anti‐CD11c (clone: N418), anti‐B220/CD45R (clone: RA3‐6B2), anti‐I‐A/I‐E (clone: M5/114.152), anti‐CD4 (clone: RM4‐5 and GK1.5), anti‐pDCA1 (clone: 927), anti‐CD8α (clone: 100712), anti‐TCR‐β (clone: H57‐597), all from Biolegend, and a viability marker (LIVE/DEAD^®^ Fixable Near‐IR Dead Cell Stain Kit, Life Technologies, Eugene, OR). For MACS cell purification, we used anti‐CD11c (cat. 130‐052‐001), anti‐CD4 (cat. 130‐049‐201), and anti‐CD8 (cat. 130‐049‐301) microbeads (Miltenyi Biotec Bergisch Glabach, Germany). For the in vitro functional assays, DC subpopulations were purified from spleens by MACS. CD11c cells were then sorted by FACS (BD FacsAria III) using APC/Cy7‐labeled anti‐B220, PercP‐labeled anti‐I‐A/I‐E, APC‐labeled anti‐pDCA1, PE‐labeled anti‐CD11b and PE/Cy7‐CD8a. We obtained 90.9 ± 1.35% purity for CD8αcDCs, 95.1 ± 1% for CD11b cDCs and 94.± 0.3% for pDCs. All cells were acquired using a FACS‐LSRFortessa according to BD bioscience protocols and analyzed by FlowJo software version 9.8.1.

### Cytokine measurements

Cytokine production was measured in cell culture supernatants and serum. ELISA specific for murine IFN‐γ (R&D Systems) was performed, according to manufacturer's instructions. IL‐27p28 was measured using mAbs generated in our laboratory as previously described 45. In all ELISAS, biotinylated detection Abs were used followed by avidin‐HRP (Biolegend). All absorbance reads are made at 450 nm, using a 96‐well plate spectrophotometer (VERSAmax, Molecular Devide).

### Type I IFN bioassay

Type I IFN activity was measured by a cytopathic effect reduction assay as described in Figure 7D 46.

### Statistical analysis

Statistical analysis was performed with Instat data analyzer and Prism 5 (Graphpad Software, La Jolla, CA) using non‐parametric tests (Kruskal–Wallis or Mann–Whitney), parametric test (Bonferroni's multicomparison), and Log‐rank Test for survival curve.

## Author Contributions

MG and RGM contributed equally to the design and performance of experiments, analyzed data, and wrote the paper, CU contributed to the development of the project and critically read the paper, ND designed and performed FACS sorting experiments, AS and TM performed experiments and analyzed data; BR suggested the use of C57Bl/6 TLR7 Kos, and RGM, JVS, and JPC conceived the research project, designed experiments, and wrote the paper.

## Conflict of Interest

The authors declare no financial or commercial conflicts of interest.

## Supporting information

Additional supporting information may be found in the online version of this article at the publisher's web‐site

**Figure S1**. Only conventional DCs are able to activate allogeneic T cell response in vitro.
**Figure S2**. LDV infection affects cDCs in a TLR7‐dependent process.
