# Supplementary figures and images for: Mouse nidovirus LDV infection alleviates graft versus host disease and induces type I IFN‐dependent inhibition of dendritic cells and allo‐responsive T cells
Source: Immun Inflamm Dis. 2017 Apr 4;5(2):200–13. doi: 10.1002/iid3.157 (PMC5418140; doi:10.1002/iid3.157)

Supplementary Figure 1

A

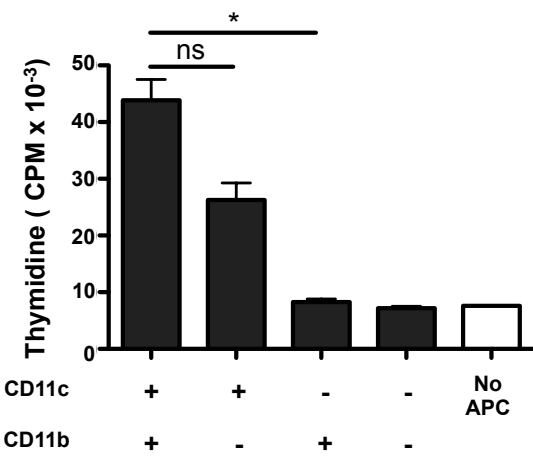

B

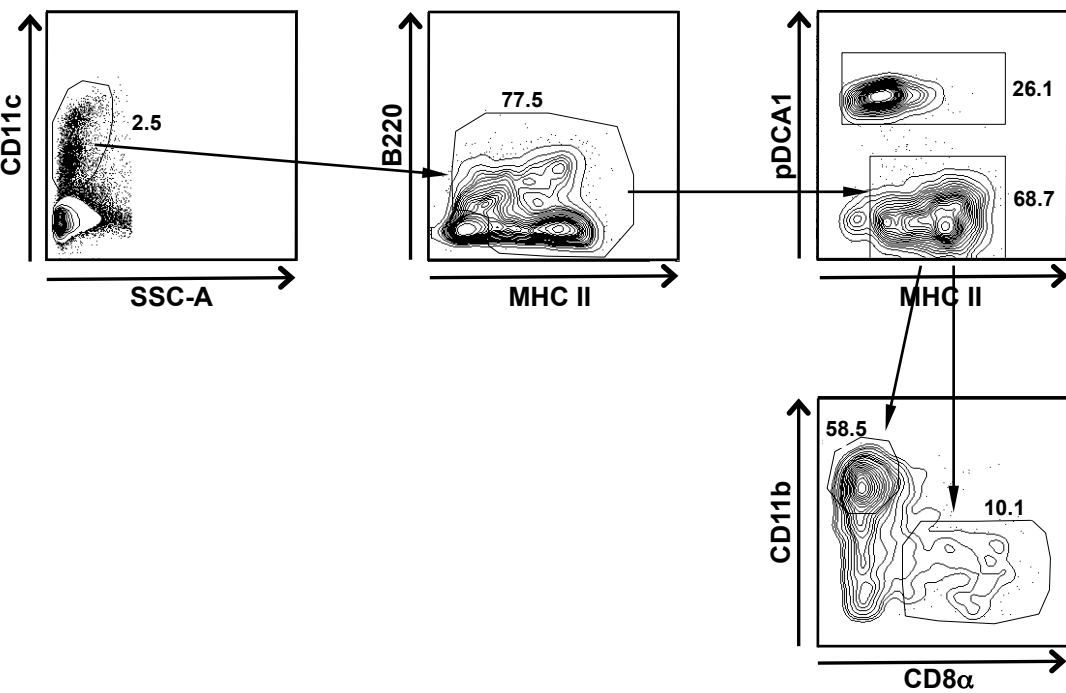

Supplementary Figure 2

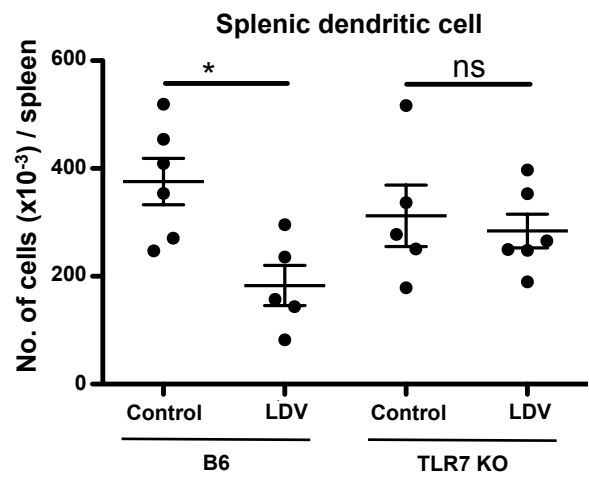

Supplement: Supplementary file 1 — Figure S1. Only conventional DCs are able to activate allogeneic T cell response in vitro. Figure S2. LDV infection affects cDCs in a TLR7‐dependent process. [file IID3-5-200-s001.pdf]
